# The sexual behavior of young people living with a disability: Findings from the KAP study in Northern Uganda

**DOI:** 10.3389/frph.2023.1065437

**Published:** 2023-03-15

**Authors:** Paul Bukuluki, Symon Peter Wandiembe, Peter Kisaakye, Victor Kiwujja, Christine Kajungu, Wilberforce Mugwanya, Shakira Nabakooza, Cyprian Anyii

**Affiliations:** ^1^ Department of Social Work and Social Administration, Makerere University, Kampala, Uganda; ^2^ Department of Statistical Methods and Actuarial Sciences, Makerere University, Kampala, Uganda; ^3^ Department of Population Studies, Makerere University, Kampala, Uganda; ^4^ Department of Sexual and Reproductive Health and Rights, United Nations Population Fund, Kampala, Uganda

**Keywords:** sexual behaviour, young people, disability, Uganda, northern Uganda

## Abstract

**Introduction:**

Young people living with disability form one of the most vulnerable population groups globally. There is limited information on the use of SRH services among young people living with a disability.

**Methods:**

This analysis is based on household survey data among young people. Drawing on a sample of 861 young people living with a disability (15 -24 years), we investigate the sexual behaviour, and identify the risk factors associated with sexual behavior of young people living with a disability. Multilevel logistic regression was used.

**Results:**

Results indicate that risky sexual behaviour was associated with alcohol consumption (aOR = 1.68; 95%CI: 0.97, 3.01), having limited knowledge of HIV and STI prevention methods (aOR = 6.03; 95%CI: 0.99, 30.00), and low life skills (aOR = 4.23; 95%CI: 1.59, 12.87). The odds of not using a condom at last sex were significantly higher among in-school young people than out of school young people (aOR = 0.34; 95%CI: 0.12, 0.99).

**Discussion:**

Targeted interventions aimed at reaching out to young people living with a disability should consider their sexual and reproductive health needs, barriers, and facilitators. Interventions can also promote self-efficacy and agency of young people living with a disability in making informed sexual and reproductive health choices.

## Introduction

The Convention on the Rights of Persons with Disabilities (CRPD) advocates for enjoyment of all human rights and fundamental freedoms among people living with disabilities (1). Disability occurs as a result of an impairment of cognitive, developmental, emotional, physical, mental, sensory or a combination of all that affects one to fully participate in activities (2). Goal Three of the Sustainable Development Goal (SDG) calls for universal access to health including access to sexual and reproductive health (SRH) services and information (3–5).

Young people living with a disability (YPWD) form one of the most vulnerable population groups globally (6). YPWD form a significant proportion of young people in developing countries; with nearly 80 percent of 180–220 million YPWD globally living in developing countries (7, 8). In Uganda, the recent 2016 Uganda Demographic and Health Survey reports that 16% of young people aged (10–19 years) were living with a disability (9). Access to SRH services among YPWD is a public health issue (10). There is limited information on the use of SRH services among this population group (11). While sexual behavior may be regarded as a universal aspect for every human being (12, 13), some literature has pointed to misconceptions surrounding sexual behavior among YPWD (14). For example, there is a view that YPWD do not engage in sexual activity or that they are asexual (15) or that having quality sexual behavior is less important compared to providing medical attention or rehabilitation needed for them (16). These views assume that YPWD do not need information regarding prevention risky sexual behavior or how to maneuver around it.

The quality of life for every individual including YPWD is an important dimension (17), which improves wellbeing (18). Further, YPWD can have unsafe sex (19), or engage in sexual behavior, but may miss the necessary skills, negotiation power and information to engage in safer sexual activity (8, 20). As a result, YPWD are often marginalized, discriminated, and relegated to the background or neglected when it comes to accessing SRH services (21–24). Moreover, health workers often miss some information regarding the sexual behavior of YPWD and are unable to advise them on some prevention strategies against STIs (25). YPWD are unable to receive SRH services or information in the form of contraception to prevent unwanted pregnancies (26), or even prevent STIs because of the misconception that YPWD do not have sex (27). Other challenges associated with limited information or services among YPWD include physical barriers (28), transport challenges (29), long waiting times (30), lack of confidentiality (31), need for an escort (32) and disability-related stigma (33) or negative attitudes (34). While these challenges are common across the board, they tend to be exacerbated among YPWD because they are categorically different, and with different needs. For example, the young people with mental disability would have different requirements from those with physical disability.

In general, given the challenges associated with limited access to SRH services, YPWD tend to engage in risky sexual behavior with far reaching negative consequences (35). For example, YPWD tend to experience child marriage (35), unintended pregnancies (36), induced abortion (21), sexually transmitted infections (STIs) including HIV (37) because of risky sexual behavior. While access to SRHR services is a general problem, YPWD tend to be more vulnerable than their counterparts. YPWD are more likely to engage in risky sexual behavior or abused than young people who are not living with a disability (38).

Most interventions are tailored towards young people without taking into consideration the vulnerable groups such as YPWD (39). This paper therefore seeks to understand the sexual behavior of YPWD, and the determinants associated with sexual behavior of YPWD. The results from such analyses can inform the design of interventions on reproductive health among YPWD. Moreover, the results can contribute to improving the quality of SRH services among YPWD.

## Methods

The current analysis is based on household survey data collected for the baseline knowledge, attitudes and practices (KAP) study of the UNFPA's supported program on Advancing Sexual Reproductive Health and Rights (ANSWER) in Northern Uganda. The study population was young people (15–24 years). Data were collected between August and September 2021. The household KAP survey was based on a stratified two-stage cluster design with stratification on districts, cross-stratified on the rural-urban residence. In refugee-hosting districts, cross-stratification was also done on refugee communities and host communities. In the first stage, a probability proportional to the size sample of villages was taken from each stratum. In the second stage, a probability systematic sample of households with young people (15–24 years) was taken.

The outcome variables in this study included: involvement in risky sexual behavior (RSB) in the past 12 months, that is multiple sexual partnerships, transactional sex and non-use of condoms at last sex with a non-marital partner. The explanatory variables included age, education, marital status, nationality, alcohol consumption, household wealth, religion, exposure to media, attitudes towards teenage pregnancy and condom use, knowledge of sexually transmitted infections, HIV and their prevention methods, life skills, self-efficacy, gender norms, type of disability. The assessment of attitudes toward teenage pregnancy was based on a proxy indicator, where respondents were asked to justify having a child whilst still a teenager. These included: (a) having a baby to love, (b) moving out of parent's house, (c) getting married early, (d) proving one's fertility, (e) enjoying the child/children growing up, (f) having a partner to love, and (g) proving you are a man/woman. However, we note that we are likely to miss out on the bad/negative attitudes especially from teenagers who have ever been pregnant since the response options available are only about the positive/good things.

*Household asset index* as a proxy measure for the economic wellbeing of a household was constructed through Principal Component Analysis model of household owned domestic items (radio, television set, sofa sets, mattress, solar/electricity for lighting, access to running in the house or yard), transport assets (bicycle, motorcycle, car), and productive assets (computer, mobile phones) and has an income generating activity. Generally, based on the pca scores, households were classified as having high household assets index if they had at least 6 of these 12 items assessed.

*Knowledge of preventing HIV/AIDS and STIs*, and their treatment was obtained from alpha factoring of six items. Respondents were asked to affirm the following: (a) having and being faithful to only one sexual partner is an effective way of preventing HIV, (b) a person can reduce their chances/risk of getting HIV by not having sex, (c) a person can reduce their chances/risk of getting HIV by using condoms when having sex, (d) the HIV/AIDS virus can be transmitted by mosquito bites, (e) the HIV/AIDS virus can be transmitted by supernatural means, (f) a person can become infected by sharing food with a person who has the AIDS virus, (g) a girl or boy cannot get HIV the first time she/he has sexual intercourse, and in addition to knowledge at least two other STIs in addition to HIV/AIDS, and source of their treatment.

*The score for gender and social norms* was computed from the responses to the following questions, with Likert scale options: (a) boys and girls have equal abilities, (b) Giving a bath and feeding kids are the mother's responsibility, (c) Woman's role is taking care of her home and family, (d) a man should have the final word about decisions in the home, (d) preventing pregnancy is a woman's responsibility, (a) Young people like you/me should not be allowed to use contraceptive services; (b) It is wrong for young girls who are sexually active to use contraceptives, (c) Women who use contraception may become promiscuous.

*Life skills* score for self-efficacy to avoid risky sex, including using a condom was measured by asking the respondents to affirm to the following statements: (a) I am confident I can get the person with whom I have sex to use a condom, even if he/she doesn't want me to use a condom, (b) I am confident If my partner and I do not have a condom, I can say no to sex, (e) I make smart decisions to avoid unsafe sex. *Self-efficacy* was measured by asking the respondents to affirm to the following statements: (a) I am confident if I did not want to have sex, I would be able to refuse sex with a person who has power over me, like a teacher, employer, relative, etc., (b) I am confident I can get the person with whom I have sex to use a condom, even if he/she doesn't want me to use a condom, and (c) I am confident If my partner and I do not have a condom, I can say no to sex.

*Positive attitude condom use* was measured by asking respondents to respond to the following statements: (a) condoms reduce sexual pleasure, (b) condoms can slip off a man's penis and enter a woman's body, (c) it is too embarrassing for someone like you to buy or obtain condoms, and (d) if a girls suggested to her partner to use a condom it would mean that she does not trust him.

An additional module based on the Short Set of questions developed by the Washington Group on Disability Statistics to estimate the prevalence of disabilities was also included in the Household Questionnaire. The Short Set questions address six core functional domains—seeing, hearing, communication, cognition, walking, and self-care.

The analysis is based on 861 young people living with disability, from among the 6,056 young people aged 15–24 years. These were mainly individuals with some difficulties with their sight (42%), hearing (25%), and memory (17%), and 27% had physical disabilities. We note that this sample size, based on sample size formula by Cochran (1963), is adequate for estimating of a proportion of YPWDs involved in risky sexual behaviour within the two standard errors with 95% certainty. This holds for population level proportions of at least 10% of YPWDs engaging in risky sexual behaviour.

At the bivariate analysis level, *F*-test was used to test for associations. Variables with *p* < 0.10 at the bivariate level and those contextually important (sex, age, schooling status and marital status) were included in multilevel logistic regression model for each of the three outcome variables. In addition, inverse probabilities of selection were included in the models as weights to further account for the complex sample survey design features. Analyses were executed in Stata V15. In multivariable regression models, adjusted model coefficients and their 95% confidence intervals (95% CI) were computed using the stepwise approach. Hosmer-Lemeshow test statistics were conducted to check model goodness of fitness. This study was approved by Uganda National Counsil for Science and Technology (UNCST) (HS1079ES).

## Results

### Sample characteristics of respondents

Of the 861 respondents, 45.4% were female, 44.4% were aged 20-24 years, and 50.9% were out of school (Table 1). Further, 10.2% were refugees (10.2% males vs. 10.2% females), 61.7% were Roman Catholics (60.6% males vs. 63.1% females), and 33.8% were living in a marital relationship (37.2% males vs. 29.6% females), and only 21.8% had a secondary school education (24.5% males vs. 18.5% females). Sixty-three percent reported exposure to media at least once a month, notably radios, newspapers, tabloids, television, and social media. Most of the respondents (75.8%) were from households with less than four of the 12 household assets assessed, and less than 1% were from households that had at least 10 assets. About 12% reported alcohol consumption within the last week. The distribution of age groups was similar between male and female respondents but there were slightly more females married than males (33.8% vs. 29.6%), and more males consume alcohol than females (16.0% vs. 6.4%).

**Table 1:** Sample characteristics.

	Male respondents		Female respondents		All	
	*n* = 486	%	*n* = 375	%	*n* = 861	%
**Socio-demographics**						
Age group (years)
15–17	107	32.1	131	48.6	238	39.6
18–20	189	28.6	131	22.4	320	25.8
21–24	190	39.3	113	29.0	303	34.6
Highest education level
None	36	9.8	32	11.2	68	10.4
Primary	260	57.2	240	68.1	500	62.2
Secondary	143	24.5	87	18.5	230	21.8
TVET	47	8.6	16	2.1	63	5.6
Current schooling status
In-school	159	44.8	138	54.1	297	49.0
Out of school	327	55.2	237	45.9	564	51.0
Current marital status
Married/living with a partner	146	37.2	106	29.6	252	33.8
Not in a union	340	62.8	269	70.4	609	66.2
Nationality
Ugandan	445	89.8	337	89.8	782	89.8
Refugee	41	10.2	38	10.2	79	10.2
Religion
Roman Catholic	285	60.6	239	63.1	524	61.7
Anglican/protestant	102	20.9	83	23.5	185	22.1
Moslem	63	12.1	15	4.0	78	8.5
Pentecostal /born again/evangelical	36	6.4	38	9.5	74	7.8
Household Asset Index
Low	370	75.7	296	75.9	666	75.8
Moderate	114	23.7	77	23.9	191	23.8
High	2	0.5	2	0.2	4	0.4
• Drinks alcohol	96	16.0	30	6.4	126	11.7
• Exposed to media at least once monthly	362	73.2	190	51.7	552	63.4
• Severe disability (a lot of difficulty)	101	18.8	60	15.6	161	17.3
**Sexual behaviour characteristics**						
Ever had sex	272	54.8	177	40.3	449	48.2
Age at sexual debut (ever had sex group)
<15 yr	36	11.5	25	16.9	61	13.6
15–17 yr	125	44.9	77	36.4	202	41.6
18+	71	26.9	56	36.8	127	30.7
Not sure/No Response	40	16.7	19	9.9	59	14.1
Risky sexual behaviour in last 12 months
Not sexually active	214	45.2	198	59.7	412	51.8
Sexually active - no RSB	146	31.6	127	30.7	273	31.2
Sexually active - RSB	126	23.2	50	9.7	176	17.1
• Last sex act was with a partner who is 5 years older	272	1.0	177	23.6	449	9.2
• Partner/self currently using contraceptives (including condoms)	272	34.1	177	31.6	449	33.1
• Ever been pregnant/impregnated a woman	272	26.3	177	68.8	449	–
• Prevalence of unintended pregnancies	–	–	122	40.4	122	40.4

Note: RSB means Risky Sexual Behaviour. In this study, we refer to heterosexual intercourse.

### Sexual behaviour of respondents

Of the 861 individuals, 48.2% (40.3% of females and 54.8% of males) reported having ever had sex, and 17.1% (9.7% of females and 23.2% of males) reported having been involved in risky sexual behavior (RSB) in the past 12 months preceding the survey, i.e., multiple sexual partnerships, transactional sex and no use of condom use in the last sex act with a non-marital partner. Fourteen percent (16.9% of the females and 11.5% of the males) had sex before turning 15 years of age, and 53.3% of females and 56.4% of males had sex before the age of 18 years. Equivalently, among all the YPWD, 6.6% (6.3% of the male and 6.8% of the female) and 26.6% (32.2% of the male and 22.0% of the female) had sex before the ages of 15 years and 18 years, respectively. Nearly, one in four young women and girls reported their last sexual partner in the past 12 months to have been at least five years older than them.

The current use of modern contraceptives (including consistent use of male condoms) was reported by 33.1% of the young people while 40.4% of the girls and young women reported their last pregnancy as unintended.

### Factors associated with risky sexual behavior

**Engagement in multiple sexual partnerships in the past 12 months:** The involvement in multiple sexual partnerships within the past 12 months was mostly reported by men and boys, the YPWD who consume alcohol, and those negative gender norms. A higher proportion of individuals who consume alcohol reported multiple sexual partners than the proportion of individuals who do not consume alcohol (41.9% vs. 25.8%, *F* = 4.99; pval = 0.027). *M*oreover, a significantly higher proportion of individuals with negative gender norms than that of individuals with equitable gender norms reported multiple sexual partnerships (32.7% vs. 24.0%, *F* = 2.89, pval = 0.049).

**Condom use in the last risky sex act in the past 12 months:** Results in Table 2 show that up to 32% of male respondents and 27% of female respondents did not use a condom in their most recent risky sex act. At bivariate analysis, secondary education level, knowledge of HIV prevention methods, knowledge of other STIs (in addition to HIV/AIDS), knowledge of where to obtain contraceptives (including condoms), positive gender norms, high score of life skills and self-efficacy, and unfavorable attitudes toward teen pregnancy were associated high percentages using a condom. Eighty-seven percent of respondents with secondary school education as compared to only 23.5% of those without formal education reported condom use in the last risky sex act (*F* = 7.10; pval = 0.049). Similarly, a higher percentage of individuals with good knowledge of HIV prevention methods (74.4%) used a condom as compared to 47.1% of those with limited knowledge (*F* = 3.951; pval = 0.036). More individuals (76.2%) with positive gender norms as compared to 63.0% of those with negative gender norms used a condom (*F* = 2.93, pval = 0.049), and 73.1% of individuals with high self-efficacy to avoid risky behavior as compared to 20.9% of individuals with low self-efficacy used the condom (*F* = 5.945; pval = 0.001). However, positive, or negative attitudes toward condom use did not matter.

**Table 2:** Relationship between background factors and risky sexual behavior among the YPWD.

		Had sexual debut when younger than 18 years		Used a male condom in the last sex act		Had multiple sexual partners in the past 12 months	Had RSB in the past 12 months (sexually active)	Had RSB in the past 12 months (all)^a^
	No.	%	No.	%	No.	%	%	%
Sex
Male	486	32.3	96	67.9	222	40.8	42.3	23.2
Female	375	22.0	47	73.0	137	8.0	24.0	9.7
Age group								
15–17 yrs	238	12.5	19	78.2	41	33.8	45.8	5.7
18–20	320	36.2	60	69.4	146	28.0	43.0	22.7
21–24	303	38.5	64	64.2	172	28.5	30.1	25.9
Highest level of education
None	68	15.6	2	23.5	12	37.1	37.4	6.3
Primary	500	27.7	78	59.4	216	29.2	33.6	15.9
Secondary	230	31.6	53	87.1	100	30.5	41.5	23.8
TVET	63	33.4	10	100.0	31	19.5	31.0	23.6
Current schooling status
In-school	297	13.2	48	68.7	50	34.5	53.2	9.0
Out of school	564	41.4	95	70.2	309	28.0	31.7	24.8
Marital status
Married/cohabiting	252	51.5	11	49.7	212	25.5	25.5	25.4
Not in union/Single	609	15.4	132	73.4	147	37.6	58.6	12.8
Nationality
Ugandan	782	27.7	130	68.9	335	28.4	34.7	16.9
Refugee	79	26.8	13	73.1	24	34.8	41.5	18.2
Household Asset Index
Low	666	28.7	100	61.3	272	30.3	34.2	16.8
Moderate	195	24.2	43	91.6	87	24.7	39.6	17.8
Religion
Roman Catholics	524	28.6	88	62.4	225	26.7	33.2	16.2
Protestants	259	25.4	43	84.7	102	29.6	39.6	18.0
Moslems	78	28.3	12	65.1	32	41.1	41.3	20.0
Exposure to media
No	309	29.1	43	62.1	126	24.6	27.3	13.4
Yes	552	26.7	100	73.4	233	31.3	40.1	19.1
Alcohol consumption
No	735	24.7	106	72.0	283	25.8	33.1	14.8
Yes	126	49.9	37	60.0	76	41.9	45.2	34.2
Knowledge of when to seek support to prevent pregnancies
No	366	21.9	40	63.4	118	30.1	32.0	11.5
Yes	495	32.5	103	72.1	241	28.4	37.1	21.8
Knows two other STIs apart from HIV/AIDS
No	454	20.8	67	62.9	161	27.6	33.6	13.1
Yes	407	35.5	76	76.8	198	29.9	36.7	21.7
Knows all ways of HIV prevention
No	288	18.6	26	47.1	88	20.4	22.3	7.4
Yes	573	32.5	117	74.4	271	31.3	39.5	22.4
Favourable attitudes toward teen pregnancy
Yes	239	31.4	30	47.5	105	27.8	28.0	15.3
No	622	26.3	113	75.3	254	29.4	39.7	17.7
Positive attitudes toward condom use
No	730	29.0	126	73.1	318	30.5	36.5	18.6
Yes	41	46.7	12	75.6	25	26.2	38.9	28.1
Life skills score
Low	29	18.8	4	27.1	8	20.0	20.0	2.8
Moderate	185	19.3	22	70.7	71	20.7	30.9	12.8
High	647	30.7	117	71.2	280	30.9	37.2	19.2
Self-efficacy in avoiding risky behaviours score
Low	101	21.0	7	20.9	31	28.9	29.0	8.8
Moderate	113	25.0	13	70.6	49	27.2	34.3	17.3
High	647	29.2	123	73.1	279	29.2	36.7	18.4
Gender norms score
Positive	448	22.2	72	76.2	165	24.0	36.5	14.5
Negative	413	34.0	71	63.0	194	32.7	34.4	20.1
**Type of disability**								
Sight difficulty
No	505	57.6	71	64.7	192	23.7	26.0	11.3
Yes	356	56.7	72	73.3	167	34.1	34.7	19.0
Hearing impairment
No	649	54.8	115	74.8	282	29.5	31.1	15.1
Yes	212	64.9	28	48.7	77	26.7	27.4	13.0
Physical impairment
No	624	56.9	107	66.2	299	32.6	35.4	16.9
Yes	237	58.0	36	81.9	60	17.3	16.6	8.2
Memory difficulty
No	699	58.6	118	72.5	289	28.7	29.5	14.6
Yes	162	48.8	25	52.7	47	30.6	34.4	14.2
Self-care difficulty
No	780	59.7	130	71.7	325	28.0	29.2	13.9
Yes	81	35.0	13	46.4	34	37.5	39.4	21.1
Mild mental difficulty
No	791	56.9	128	68.7	330	29.6	31.0	15.1
Yes	70	62.5	15	80.0	29	17.1	17.1	7.4

^a^ Note: Sample size as in second column. Figures do not add up to 100 percent because we are focusing on only young people involved in risky sexual behaviour.

**Involvement in risky sexual behavior (composite score) among sexually active young people in the last 12 months:** Bivariate analysis results in Table 2 show that a lower proportion of female YPWD (24.0%) engaged in RSB than the male YPWD (42.3%) (*F* = 11.06; pval = 0.001); and lower proportion of out of school YPWD (31.7%) than the proportion of in-school YPWDs (53.2%) reported RSB in the past 12 months preceding the survey (*F* = 5.55, pval = 0.019). The results also show that a higher percentage of individuals with good knowledge of HIV prevention methods engaged in risky sexual behavior as compared to those with limited knowledge (39.5% vs. 22.3%; *F* = 6.96; pval = 0.009).

**Involvement in risky sexual behavior (composite score) among all young people in the last 12 months:** Among all YPWD (sexually active and non-sexually active), only 17.2% (23.2% of male and 9.7% of female) of young people were involved in risky sexual behaviour (RSB) within the last 12 months (Table 2). Bivariate analysis presented in Table 2 show that having secondary school education, being out of school, consuming alcohol, and holding inequitable gender norms were associated with RSB within the past 12 months. Twenty percent of individuals with inequitable gender norms as compared to 14.5% of individuals with equitable gender norms were engaged in RSB (*F* = 3.19; pval = 0.045); and 34.2% of individuals who drink alcohol as compared to only 14.8% of those who do not reported RSB. Overall, being in school was protective of engagement in RSB as only 9.0% of the in-school YPWD engaged in RSB as compared to 24.8% of the out of school young people (*F* = 17.00; pval < 0.001). Taken together with results in the previous section, this implies that schooling is a protective factor to RSB but the few in-school young people who become sexually active face higher risks of pregnancy, and STIs than the out of school young people.

**Sexual debut before 18 years of age:** The results are presented in Table 2. At bivariate analysis, factors associated with early initiation of sexual debut were being male (32.2% vs. 22.0% of females; *F* = 11.25, pval = 0.004); being out of school (41.4% vs. 13.2% of in-school; *F* = 45.29; pval < 0.001); alcohol consumption (49.9% vs. 24.7% of non-alcohol consumers; *F* = 24.46; pval < 0.001); and holding negative gender norms (34.0% vs. 22.2% of individuals with positive gender norms; *F* = 9.06, pval = 0.003).

### Multivariable analysis of factors associated with risky sexual behavior

The odds of involvement in RSB relative to other sexually active young people were independently increased by alcohol consumption (aOR = 1.68; 95%CI: 0.97, 3.01) and higher among boys and men than girls and women (see Table 3). The odds of engaging in RSB was low among young people with life skills; young people who reported below average scores of life skills had more than four times the odds of engaging in RSB than those with above average scores – above vs. below average had (aOR = 0.23; 95%CI: 0.07, 0.79). Similarly, the involvement in RSB with respect to the whole population of YPWD was also driven by these same factors, in addition to age group.

**Table 3:** Multivariable analysis of factors associated with risky sexual behavior.

	Had multiple sexual partners in the past 12 months		Did not use a male condom in the last sex act		Had RSB in the past 12 months (among sexually active)	
	Crude OR (95%CI)	Adj. OR (95%CI)	Crude OR (95%CI)	Adj. OR (95%CI)	Crude OR (95%CI)	Adj. OR (95%CI)
Sex
Male	1.00		1.00		1.00	
Female	0.13 (0.06, 0.27)	**0.13** **(0.06, 0.28)**	0.78 (0.26, 2.38)	0.82 (0.22, 3.16)	0.43 (0.26, 0.71)	**0.29** **(0.16, 0.53)**
Age group
15–17 yrs	1.00		1.00		1.00	
18–20	0.76 (0.29, 1.98)	0.71 (0.27, 1.83)	1.59 (0.21, 12.5)	2.1 (0.3, 14.84)	0.89 (0.38, 2.08)	0.75 (0.32, 1.74)
21–24	0.78 (0.26, 2.37)	0.8 (0.28, 2.29)	2 (0.32, 12.5)	2.27 (0.27, 19.25)	0.51 (0.19, 1.35)	0.72 (0.33, 1.58)
Current schooling status
In-school	1.00		1.00		1.00	
Out of school	0.74 (0.26, 2.07)	1.39 (0.56, 3.49)	0.93 (0.39, 2.22)	**0.34** **(0.12, 0.99)**	0.41 (0.19, 0.88)	1.4 (0.69, 2.83)
**Marital status**						
Married/cohabiting
Not in union/Single	1.77 (0.08, 0.93)	**2.22** **(1.23, 4.01)**	0.36 (0.08, 1.52)	0.32 (0.06, 1.79)	4.16 (2.38, 7.28)	**2.16** **(1.27, 3.66)**
**Household Asset Index**						
Low
Moderate	0.75 (0.4, 1.41)		0.18 (0.06, 0.55)	**0.19** **(0.06, 0.64)**	1.24 (0.7, 2.2)	
Alcohol consumption
No	1.00		1.00		1.00	1.00
Yes	2.08 (1.08, 3.99)	1.57 (0.76, 3.26)	1.72 (0.61, 4.76)	**2.63** **(1.11, 6.19)**	1.67 (0.96, 3.02)	**1.68** **(0.97, 3.01)**
Knows all ways of HIV prevention
No	1.00		1.00		1.00	
Yes	1.33 (0.76, 2.35)		0.53 (0.24, 1.14)	**0.23** **(0.08, 0.63)**	1.38 (0.87, 2.19)	
Favourable attitudes toward teen pregnancy
Yes	1.00		1.00		1.00	
No	1.08 (0.57, 2.03)		0.3 (0.09, 1.02)	0.34 (0.07, 1.75)	1.88 (1.13, 3.12)	
Positive attitudes toward condom use
No	1.00		1.00		1.00	
Yes	0.81 (0.26, 2.5)		0.88 (0.31, 4.14)	0.24 (0.02, 2.87)	1.11 (0.43, 2.82)	
Life skills score
Low	1.00		1.00		1.00	
Moderate	1.04 (0.15, 7.43)		0.15 (0.01, 1.92)		3.73 (0.99, 18.68)	**0.23** **(0.07, 0.79)**
High	1.79 (0.29, 11.11)		0.15 (0.02, 1.32)		4.92 (0.95, 20.65)	**0.43** **(0.24, 1.09)**
Self-efficacy in avoiding risky behaviours score
Low	1.00		1.00		1.00	
Moderate	0.92 (0.25, 3.37)		0.11 (0.02, 0.71)	0.45 (0.05, 3.88)	1.54 (0.5, 4.68)	
High	1.02 (0.36, 2.87)		0.1 (0.02, 0.6)	**0.16** **(0.02, 1.03)**	1.71 (0.68, 4.26)	
Highest level of education
None	1.00		1.00		1.00	
Primary	0.7 (0.18, 2.77)		0.21 (0.01, 3.57)		1.11 (0.29, 4.21)	
Secondary	0.74 (0.18, 3.1)		0.05 (0.01, 0.19)		1.55 (0.4, 5.97)	
TVET	0.41 (0.07, 2.25)				0.98 (0.22, 4.4)	
Nationality
Ugandan	1.00		1.00		1.00	
Refugee	1.34 (0.36, 5.05)		0.81 (0.26, 2.63)		1.33 (0.59, 3.03)	
Religion
Roman Catholics	1.00		1.00		1.00	
Protestants	1.15 (0.6, 2.19)		0.3 (0.1, 0.88)		1.32 (0.76, 2.29)	
Moslems	1.91 (0.78, 4.68)		0.89 (0.16, 5.08)		1.19 (0.53, 2.65)	
Exposure to media
No	1.00		1.00		1.00	
Yes	1.39 (0.74, 2.61)		0.6 (0.13, 2.82)		1.78 (1.06, 3)	
Knowledge of when to seek support to prevent pregnancies
No	1.00		1.00		1.00	
Yes	0.92 (0.5, 1.71)		0.67 (0.24, 1.85)		1.25 (0.77, 2.05)	
Knows two other STIs apart from HIV/AIDS
No	1.00		1.00		1.00	
Yes	1.18 (0.65, 2.15)		0.59 (0.19, 1.85)		1.16 (0.7, 1.91)	
Gender norms score
Positive	1.00		1.00		1.00	
Negative	1.54 (0.87, 2.71)	1.53 (0.84, 2.78)	1.89 (0.69, 5)		0.91 (0.57, 1.47)	

For some of the individual components of RSB such as the odds of not using a condom in a high-risk sexual act were driven by alcohol consumption (aOR = 2.63; 95%CI: 1.11, 6.19), limited knowledge of HIV and STI prevention methods (aOR = 6.03; 95%CI: 0.99, 30.00), and low life skills score (or low self-efficacy) (aOR = 4.23; 95%CI: 1.59, 12.87). The odds of not using a condom was significantly higher among in-school young people than out of school young people (aOR = 0.34; 95%CI: 0.12, 0.99).

## Discussion

Our findings demonstrate that on average about two in ten YPWD engage in high risky sexual behavior such as multiple sexual partnerships, transactional sex and non-use of condom in the last sex act with a non-marital partner. Studies conducted in other low- and middle-income countries have found similar results that point to risky sexual behaviour among YPWD (20, 39–42). For example, Oladunni (2012) found that about a third of adolescents with a disability in Nigeria had multiple sexual partners at a time and engage in multiple sexual relationships and about 12 percent do not take any action to prevent pregnancy.

Results show early initiation of sex before 15 years (sexual debut) and by the age of 18 years more than a quarter of YPWD have had sex, and cross-generational sex was high among female YPWD. These results are in conformity with those from other studies in Africa and from other low-income countries (21, 41). For example, Oladunni (2012) found that more than half of adolescents with a disability had sex by the age of 15 years. Similarly, a study conducted in Ghana established that young people with hearing and vision loss had a higher likelihood of engaging in casual sex (42). This further reemphasizes the need to prioritise and integrate disability in programming for SRHR (43).

Consistent use of male condoms was low among YPWD, and this may explain the very high rate of unintended pregnancies that affects four in five girls and young women. Results confirm those of other studies conducted in relatively similar contexts in Africa and low-income countries (36). For example, in a study carried out in Ethiopia among YPWD, Kassa and colleagues (2014) found that only a third of them used contraceptives (21).

Individuals who consume alcohol had almost twice the odds of engaging in RSB than those who do not. These results are similar to a number of studies that underscore alcohol consumption as a risk factor for engagement in risky sexual behaviour for YPWD (44–46). For example, Marshal (2014) found that alcohol use and other risk-taking behaviours and risky sexual behaviour emerge in adolescence and tend to cluster together.

## Conclusion

This study has demonstrated that YPWD are sexually active and engage in sex as early as 15 years. The YPWD are at risk of unintended pregnancies and acquiring of sexually transmitted infections, and associated consequences. The risks are heightened by low life skills, limited agency, and alcohol consumption. The study underscores the importance of schooling and access to sexuality education. This means policies and programmes for government and non-government entities should be intentional in promoting access and completion of schooling for YPWD. This further emphasizes the need to address barriers of access to education for YPWD including social and gender norms that create stigma and downplay education for them and the other structural barriers related to infrastructure, skilled human resources especially teachers and other service providers as well as the required equipment. Interventions designed to promote safe sexual practices and negative SRH outcomes should integrate addressing alcohol as a risk factor. The results also highlight the need for interventions targeting YPWD starting early in their adolescence to ensure they have the appropriate sexuality education, life skills and agency to make informed choices related to engaging in safe sex to prevent undesirable reproductive health outcomes. The YPWD should also be engaged in co-design of interventions and services designed to meet their SRH needs and to promote safe sexual practices.

## Data Availability

The original contributions presented in the study are included in the article/**Supplementary Material**, further inquiries can be directed to the corresponding author/s.
